# Defining the area of mitoses counting in invasive breast cancer using whole slide image

**DOI:** 10.1038/s41379-021-00981-w

**Published:** 2021-12-11

**Authors:** Asmaa Ibrahim, Ayat G. Lashen, Ayaka Katayama, Raluca Mihai, Graham Ball, Michael S. Toss, Emad A. Rakha

**Affiliations:** 1 Division of Cancer and Stem Cells, School of Medicine, University of Nottingham Biodiscovery Institute, University Park, Nottingham, UK; 2 Department of Pathology, Faculty of Medicine, Suez Canal University, Ismailia, Egypt; 3 Department of Pathology, Faculty of Medicine, Menoufia University, Shebin El Kom, Egypt; 4 Diagnostic Pathology, Gunma University Graduate School of Medicine, Maebashi, Japan; 5 Department of Pathology, Queen Elizabeth University Hospital, 1345 Govan Rd, Glasgow, G51 4TF UK; 6 John van Geest Cancer Research Centre, School of Science and Technology, Nottingham Trent University, Nottingham, UK

**Keywords:** Preclinical research, Laboratory techniques and procedures

## Abstract

Although counting mitoses is part of breast cancer grading, concordance studies showed low agreement. Refining the criteria for mitotic counting can improve concordance, particularly when using whole slide images (WSIs). This study aims to refine the methodology for optimal mitoses counting on WSI. Digital images of 595 hematoxylin and eosin stained sections were evaluated. Several morphological criteria were investigated and applied to define mitotic hotspots. Reproducibility, representativeness, time, and association with outcome were the criteria used to evaluate the best area size for mitoses counting. Three approaches for scoring mitoses on WSIs (single and multiple annotated rectangles and multiple digital high-power (×40) screen fields (HPSFs)) were evaluated. The relative increase in tumor cell density was the most significant and easiest parameter for identifying hotspots. Counting mitoses in 3 mm^2^ area was the most representative regarding saturation and concordance levels. Counting in area <2 mm^2^ resulted in a significant reduction in mitotic count (*P* = 0.02), whereas counting in area ≥4 mm^2^ was time-consuming and did not add a significant rise in overall mitotic count (*P* = 0.08). Using multiple HPSF, following calibration, provided the most reliable, timesaving, and practical method for mitoses counting on WSI. This study provides evidence-based methodology for defining the area and methodology of visual mitoses counting using WSI. Visual mitoses scoring on WSI can be performed reliably by adjusting the number of monitor screens.

## Introduction

Histological grade of breast cancer (BC) is a strong prognostic and predictive factor for disease behavior and outcome^1^. Mitotic count as a component of grade reflects the rate of tumor proliferation and aggressiveness^2–5^. However, the level of concordance of mitotic count among pathologists remains low^6–8^. Such discrepancy is not only attributed to the variability in pathologists’ performance but also reflects the subjectivity and variation in methodologies used for the assessment of mitotic scores.

Mitotic scores often represent the highest proliferative activity of the tumor, and they are obtained by counting mitotic figures within hotspots (areas showing the highest number of mitoses within the tumor)^9,10^. When using light microscope, it is recommended that mitotic figures are counted in ten high-power fields (HPFs) in hotspots^11^. Different microscopes have different field areas, which vary widely (they range from 1.26 to 3.74 mm^2^ per 10 HPFs)^12^. To achieve consistency of scoring when using different microscopes, tables detailing mitotic cut-offs per each field area are published^12^. Changes of mitotic cut-offs rather than standardizing the area by adjusting the number of the HPFs accordingly are currently recommended^11–13^, despite evidence indicating that using a fixed area size is more reliable for mitotic counts in BC^14–20^. The approach toward standardizing the area for counting mitoses rather than the cut-offs per microscope field diameters has already been adopted in several other tumors such as melanoma^21,22^ and gastrointestinal stromal tumors^23^.

Although the recent introduction of whole slide image (WSI) technology in primary histopathology reporting has several advantages^24^, it can make visual assessment of mitoses more challenging^25^. Identification of mitotic figures on WSI is difficult due to loss of some of the fine details of mitoses resulting from the lack of fine-tuning ability^26^. Although this could be overcome by using higher quality scanners with focus stacking (Z stacking) functionality, the time, cost, and the image storage capacity make the availability of such options challenging in routine practice. Moreover, the methodology for defining the area for measurement on the screen is still ambiguous^27,28^. One HPF on the conventional light microscope is not equivalent to a digital HPF^28^, while the microscopic resolution is dependent on the objective and ocular lens only, the digital resolution is more complicated with additional influencing factors including the scanner objective lens magnification, the resolution of the digital camera sensor, viewing software, and the display monitor characteristics^29^. Although most the available imaging viewing software can generate absolute area size on the monitor screens, the practical application still face challenges with standardization of the methodology. In addition, the use of different combinations of scanners, imaging viewing software, and monitor screens of variable features in routine practice makes this task more challenging. Even though several studies have shown high overall diagnostic concordance between both platforms^27,30–32^, more standardization of mitotic count methodology using WSI is needed to improve the consistency of its assessment and to provide guidelines when applying artificial intelligence (AI) for automatic scoring of mitosis.

In this study, we aimed to provide evidence-based data to define the optimal area for counting mitoses in terms of the geographical distribution within the tumor and the best practical and accurate methodology for counting mitoses using WSI.

## Materials and methods

### Study cohort

**Fig. 1:**
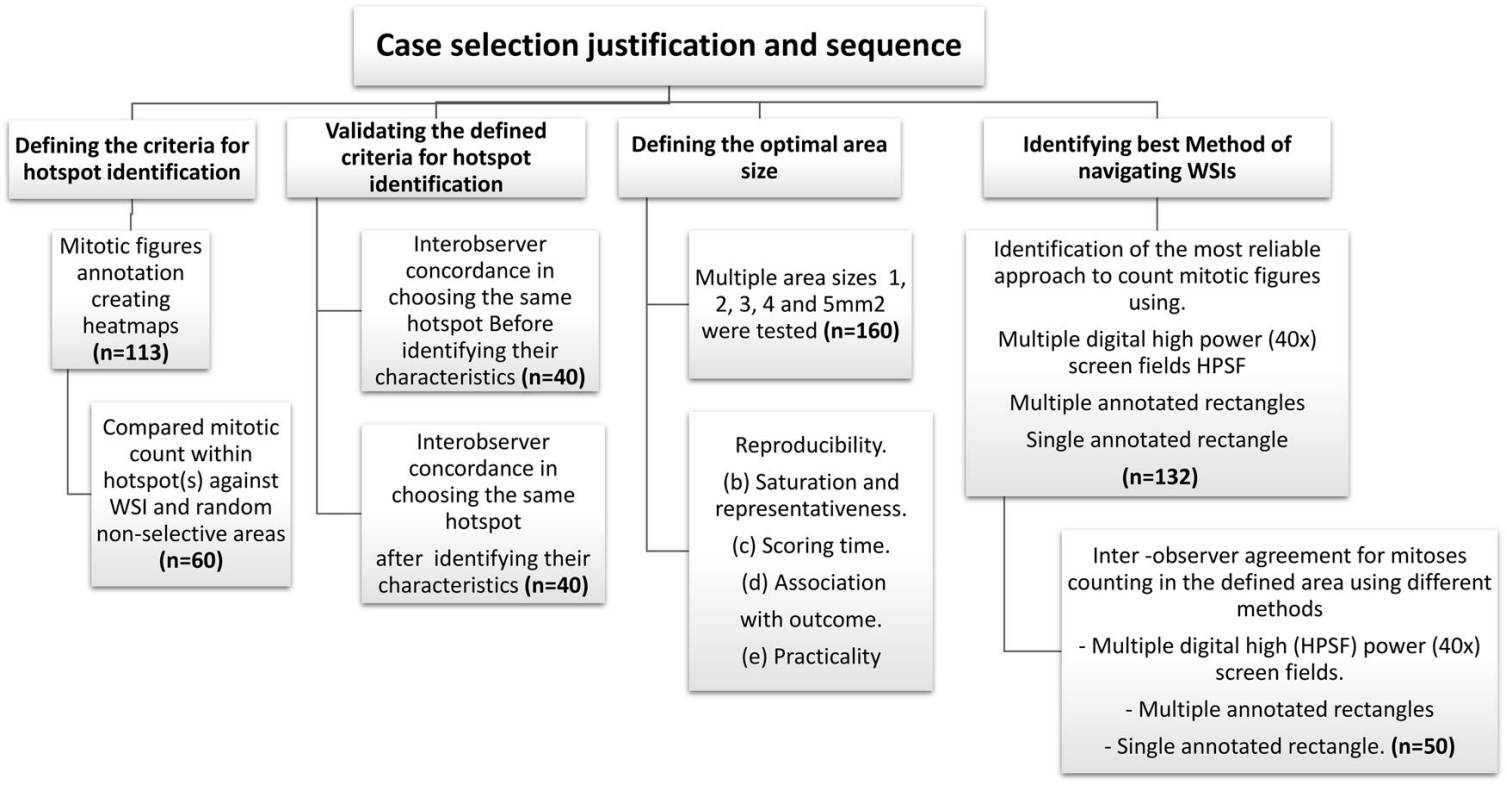
Case selection flowchart. Overview of the studied cases selection.

This study was conducted on a cohort primary invasive BC (*n* = 595). A chart outlining an overview summary of the cases selected is provided in Fig. 1.

The clinicopathological data including the molecular subtypes of the cases included in the study were available as previously described^33–35^. Tumors were classified into molecular subtypes based on the expression of estrogen receptor (ER), progesterone receptor (PR), and human epidermal growth factor 2 (HER2) as follows: (i) ER+ and/or PR+ and HER2–, (ii) HER2 enriched (ER_– and/or PR– and HER2+), and (iii) triple negative (TNBC).

Four-µm-thick, full-face tumor sections stained with hematoxylin and eosin (H&E) were used. The slides were scanned at ×40 magnification using a high throughput slide scanner (Pannoramic 250 Flash III; 3DHISTECH, Budapest, Hungary), and the images were viewed using Case Viewer software (version 2.2.0.85; 3DHISTECH) on a full-screen panel (27 inches with resolution 2560 × 1440 pixels).

### Defining the criteria for hotspot identification

To define the criteria for selecting the representative area for mitotic counting on WSIs, the mitotic figures in 113 cases were annotated manually in the entire WSI to create heatmaps. One observer screened each WSI twice for mitotic figures. Slides were examined in a systematic manner at ×40 magnification. The whole tumor area within the WSIs was examined starting from the upper left corner, moving in a Z-shaped manner to the lower right corner. All figures demonstrating the morphological criteria of true mitoses were annotated. To ensure the specificity of annotation by the first observer, all the cases were reviewed by a second observer to confirm the nature of the annotated structures (i.e., mitotic or nonmitotic cell). Only evident mitotic figures were considered, and the agreement was achieved by consensus. 56,169 mitotic figures were agreed between the two observers as true mitotic figures.

Areas with the highest numbers of mitoses (hotspots) in each slide were selected and zoomed out at ×0.5 magnification to attain a general overview of their characteristics regarding distribution (location), the relative increase in the tumor cell density, pattern of tumor growth, mitotic counts, relation between hotspots with areas of tumoral necrosis, DCIS, presence of central necrosis and fibrosis, tumor infiltrating lymphocytes (TILs), and tumor border either pushing or infiltrative.

**Fig. 2:**
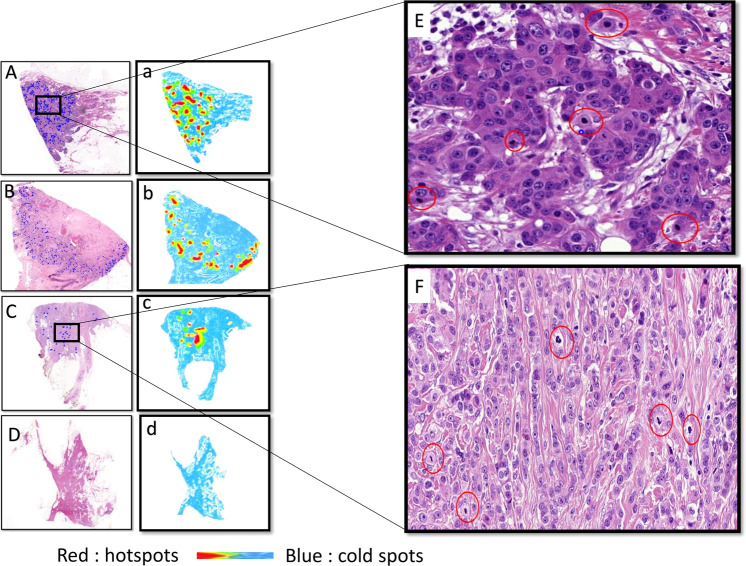
Distribution of the hotspot within analyzed cases. Upper case: original hematoxylin and eosin (H&E) at ×0.5 digital magnification. Lower case: the corresponding heatmap images at ×0.5. The blue dots are indicative of annotated true mitotic figures in H&E images. **A** Mitotic figures distributed all over the tumor (scattered) at ×0.5 digital power (H&E). **a** Heatmap of the corresponding H&E image showing mitotic figures distributed all over the tumor at ×0.5 digital power. **B** Mitotic figures at the periphery of the tumor at ×0.5 digital power (H&E). **b** Heatmap of the corresponding H&E image showing mitotic figures distributed at the periphery of the tumor at ×0.5 digital power. **C** Mitotic figures at the center of the tumor at ×0.5 digital power (H&E). **c** Heatmap of the corresponding H&E image showing mitotic figures distributed at the center of the tumor at ×0.5 digital power. **D** Tumor image showing no hotspot at ×0.5 digital power (H&E). **d** Heatmap of the corresponding H&E image showing no hotspot at ×0.5 digital power. **E** Inset images of mitotic figures distributed in scattered pattern at ×20 digital power. **F** Inset images of mitotic figures distributed in central pattern at ×20 digital power.

The distribution of hotspots in each case was determined using heatmaps generated by the annotated true mitotic figures. The hotspot distribution was classified as either peripheral (at or close to the invasive tumor fronts), central, scattered/dispersed (hotspots evenly distributed all over the tumor including both peripheral and central areas), or no hotspot could be detected where only a few separated mitotic figures present (Fig. 2). Stromal TILs were evaluated based on the International Immuno-Oncology Biomarker Working Group guidelines for invasive breast carcinoma^36^. Central fibrosis refers to central acellular zones occupied by fibrotic tissues, whereas central necrosis refers to central acellular zones occupied by necrotic tissues, based on the following criterion: necrosis/fibrosis extends for 1 mm or more over the cross-section of tumors, with abrupt transition between necrosis/fibrosis and viable tumor cells, no evidence of squamous, osseous, or cartilaginous metaplasia and of matrix-producing features.

The tumor cell density was evaluated subjectively within the areas of interest (hotspots) compared to the cellularity in other tumor areas on the same slide taking in consideration tumor cell overlapping, cellular cohesion, spacing, morphological pattern, and intervening stroma. The pattern of tumor growth was visually assessed by one observer and according to the predominant pattern (>90%), it was classified into one of the following: sheets, nests and trabeculae, tubular, cribriform, (single files), or papillary patterns (Supplementary Fig. S1). Mitotic counts were recorded as number of mitoses within the hotspots. A list of morphological criteria for identifying hotspots was proposed from this investigation (Tables 1 and 2 and Supplementary Tables S1 and S2).

**Table 1:** Characteristics of mitotic hotspots in breast cancer cases using WSIs.

Features	*N* (%)
*Relative increase in tumor cell density (cellularity) compared to surrounding areas*	
Relative increase in tumor cell density	88 (77.9)
No increase in tumor cell density	25 (22.1)
*Distribution of mitotic hotspots*	
Peripheral	39 (34.5)
Scattered	54 (47.8)
Central	9 (8.0)
No hotspot	11 (9.7)
*Hotspot next to DCIS*	
Yes	12 (11.2)
No	95 (88.8)
*Hotspot next to necrosis*	
Yes	13 (81.3)
No	3 (18.7)
*Molecular subtypes*	
ER+ and/or PR+ and HER2–	85 (75.2)
HER2 enriched	7 (6.2)
Triple negative (TNBC)	17 (15)

**Table 2:** Refined criteria for hotspot identification.

Existing data	Additional characterization
Hotspots are located at the peripheral invasive part of the tumor	Nearly half of the cases showed scattered distribution all over the tumor (at both peripheral and central areas)
Hotspots are located at the most cellular areas	Hotspots are associated with relative increase in tumor cell density
	Majority of cases with central necrosis or central fibrous scar will show peripheral distribution of hotspots, while cases without central necrosis or fibrosis are likely to have scattered, central, distribution or no hotspot at all
	Cases with peripheral or scattered hotspot showed sheets, nests or trabeculated patterns
	Cases with central hotspots have tubular and single file patterns
	Hotspots with high mitotic count are associated with sheeted (97%), nested and trabeculated pattern (84%), while hotspots with low mitotic count are associated with single files (74%), papillary (100%), and tubular patterns (50%)
	Hotspots with high mitotic count are associated with peripheral (92%) and scattered distribution (87%), while hotspots with low count are associated with central distribution (67%)

To validate the defined criteria for hotspot identification (Tables 2 and 3), we assessed the effect of the studied characteristics on identifying mitotically active areas using low digital power.

**Table 3:** Applied technical points for mitosis counting using WSI.

1	Tissue thickness of 4 µm is recommended
2	Scan the whole slide at low-power ×0.5 digital magnification for areas of high tumor cell density
3	It is mostly expected that: Majority of cases with central necrosis or central fibrous scar will show peripheral distribution of hotspots, while cases without central necrosis or fibrosis are likely to have scattered, central, distribution, or no hotspot at all Tumors arranged in sheets, nests, and trabeculae are likely to have peripheral or scattered distribution of hotspots that have been correlated with high mitotic count Tumors arranged in tubular, papillary, and single file patterns are likely to have central distribution of hotspots that have been correlated with low mitotic count If a tumor has mixed patterns, it is most likely that the highest number of mitotic figures will be associated with sheet, nests, and trabeculated patterns
4	Zooming in and out in these areas to ensure they contain enough mitotic figures
5	In tumors with homogeneous cell density either low or high, screening should be done in a systematic manner from the periphery to involve the whole slide
6	As the number of mitotic figures increases, it becomes easier to pick up mitotic figures at low power
7	A lot of factors affect the visibility of mitotic figures using digital images, like the quality of staining and scanning, crush artifact, and the fixation of the tumor
8	Tumors where the number of mitotic figures is very high, mitotic figures become distributed all over the tumor, and there is no agreement on where to start counting mitoses
9	When counting mitoses, it is a matter of preference and time availability to annotate mitotic figures to avoid confusion

We measured the level of agreement between two pathologists, before and after applying the criteria using 40 cases. Two pathologists used a copy of the same WSI, and each of them separately annotated mitotic hotspot as region of interest using the circle annotation tool of Case Viewer software. Each observer was blinded to the results of the other observer. The agreement between them was considered when these two circles overlapped or intersected (Supplementary Fig. S2). The degree of agreement was assessed statistically using Cohen’s Kappa test.

**Fig. 3:**
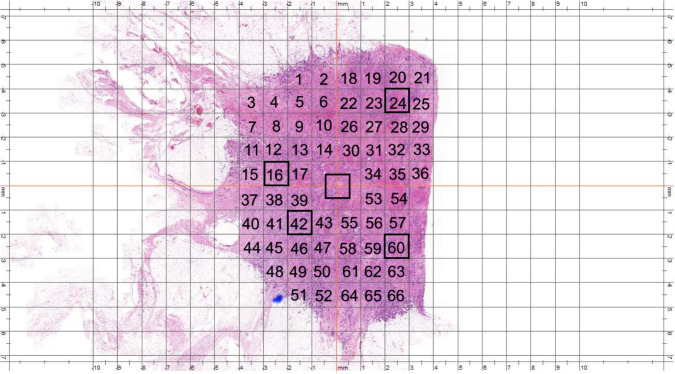
Selection of random areas using WSI. Hematoxylin and eosin (H&E) at ×0.5 digital magnification, the tumor is hypothetically divided into four quadrant and, a grid was drawn (with squares of 1 mm^2^ area size) in each quadrant and another one was drawn in the central part of the tumor. Each small square was assigned a number. Numbers were randomly ordered using free access random number generator https://stattrek.com/statistics/random-number-generator.aspx, and the first 5 numbers in the generated list in each quadrant were chosen to count mitoses within them. This was designed to represent a non-selective manner of counting mitoses in area of 5 mm^2^ in total (a total of 5 mm^2^).

To test the hypothesis that counting in hotspots represents the highest proliferative activity within the tumor, we have compared the average mitotic count in hotspots with the average mitotic count in the WSI and with mitotic counts in randomly selected areas. To record mitotic counts in randomly selected areas, the tumor on WSIs (*n* = 60) was divided into four quadrants. A grid was drawn (with squares of 1 mm^2^ area size) in each quadrant and another one was drawn in the central part of the tumor. Each small square was assigned a number. Numbers were randomly ordered using free access random number generator (https://stattrek.com/statistics/random-number-generator.aspx) and the first 5 numbers in the generated list were chosen to count mitosis within them. This was designed to represent a non-selective manner of counting mitoses in area of 5 mm^2^ in total. Figure 3 shows the selection of these random areas.

### Defining the optimal area size

The optimal area for counting mitoses within hotspots was evaluated by counting mitoses at ×40 digital magnification in multiple annotated areas including 1, 2, 3, 4, and 5 mm^2^ areas. Five rectangles, each measuring 1 mm^2^, were annotated separately in selected hotspots, avoiding areas of very low cellularity, necrosis, DCIS, tissue artifact, or areas out of focus on WSI. All cases were scored by three observers using the same protocol; the first observer scored all cases, and the other two scored 25% of the cases. The optimal size of the area for mitotic count evaluation was determined based on the level of reproducibility, scoring time, association with outcome, practicality (as determined subjectively by the observers), and level of saturation defined as the area beyond which the increase in mitotic count, if any, is not statistically significant. The latter was determined using the non-parametric, Mann–Whitney *U* test, to assess the two-level differential mitotic counts against the baseline (area size).

### Method of navigating WSIs when counting mitoses

To select the most reliable approach for counting mitotic figures on WSI, we compared three methods including (1) multiple display screens at ×40 magnification (multiple digital high-power (×40) screen fields (HPSFs)) equivalent to of 3 mm^2^ area (refer to Table 6 for how to calculate the number of HPSFs). (2) counting within a pre-annotated (3 mm^2^) as a single area. (3) counting within a pre-annotated (3 mm^2^) as multiple separate rectangles (non-adjacent small areas in different hotspots to avoid areas with low cellularity or artifacts that collectively equivalent to 3 mm^2^. Measurement of accuracy is challenging as no ground truth is available to compare each method against. We have checked the accuracy by annotating and re-assessing some of the figures that were detected by one method and missed by the others, and they were agreed by the two observers to be true mitotic figures; high concordance was used as evidence of acceptance. One of the main aims for the choice of the method is the reproducibility of the technique, as well as the consistency and concordance of scoring. Other variables include time, pathologist’s preferences, matching with the existing guidelines and current practice.

The number of HPSFs was determined by the viewing area size only, excluding the toolbars, menu bars, and any other annotation windows. The size of the viewing areas was calculated, and the number of the screens (HPSFs) was determined to produce a 3 mm^2^ area. The effect of different variables (display monitor size, screen resolution (measured in pixel density), different scanner types and viewing software) were tested on the size of the area on the WSI, by changing one variable while fixing the others and identifying which of them had the major impact. For this aim, cases were scanned by three scanners Philips IntelliSite Ultra-Fast Scanner, and Leica Aperio AT2 scanners were used in addition to Pannoramic 250 Flash III: 3DHISTECH with the relevant viewer software including IMS Philips, Aperio Image Scope and Case Viewer, respectively.

### Statistical analysis

All statistical analyses were performed using SPSS ver.26. The correlations between categorical variables were analyzed by *χ*^2^ test. The differences between the two independent groups were compared by Mann–Whitney *U* test. The degree of interobserver agreement was assessed by use of the intraclass correlation coefficient (ICC) for continuous data. Cohen’s statistic was used to assess the concordance between two observers for categorical variables while Fleiss Kappa was used for more than two variables/scores. Univariate Cox Regression model was used for outcome analysis against the BC-specific survival using the continuous mitotic score within different area size.

For all tests, *P* < 0.05 (two-tailed) was considered statistically significant.

## Results

### Defining hotspots

Table 1 summarizes the identified morphological characteristics of hotspots using WSIs.

The most frequently seen hotspot distribution pattern was the scattered pattern, accounting for 48%. Cases showing regional variability of mitotically active areas represented 42% and these either had a peripheral pattern in 34% or central in 8%, while 10% of the tumors showed no hotspots with only few separated mitoses without any clustering, which was determined subjectively by visual assessment. Central patterns were associated with lower mitotic counts in general, whereas peripheral and scattered patterns were associated with higher mitotic counts per the defined area (Supplementary Table S2).

In all, 85% of cases with a peripheral hotspot distribution showed either central necrosis or a central fibrous scar, while the majority of cases with scattered or central patterns showed neither central necrosis nor fibrous scar (76% and 82% of cases, respectively). Hotspots were frequent next to areas of necrosis (81% of cases with necrosis) but not adjacent to DCIS (11% of the cases with DCIS). No significant correlation was found between the distribution of hotspots and the presence of TILs or type of tumor border.

The relative increase in tumor cellularity within hotspots was one of the most significant parameters and was observed in 78% of cases.

Relative increase in tumor cell density was associated with peripheral, scattered, and central hotspot distribution (90%, 83%, and 55%, respectively).

In most cases with peripheral and scattered hotspot distribution, tumor cells were arranged in sheets, nests, or trabeculae, whereas the majority of cases with a central hotspot distribution showed tubular and single file patterns.

Hotspots with a high mitotic count were associated with sheeted, nested, and trabeculated patterns, while hotspots with lower counts were associated with single file, papillary, cribriform, and tubular patterns (*x*^2^ = 60.45, *P* < 0.001).

The mitotic count within the hotspot, the relative increase in tumor cell density, and the pattern of tumor growth, all showed a statistically significant correlation with hotspot distribution (*P* < 0.001) (Supplementary Table S2).

There is a statistically significant correlation between the distribution of hotspots and the presence of central necrosis with the molecular subtypes (*x*^2^ = 19.19, *P* = 0.024) and (*x*^2^ = 10.285, *P* = 0.016), respectively. Peripheral distribution and central necrosis were associated with triple-negative BC.

### Interobserver concordance in choosing the same hotspot

Table 2 shows a summary of additional and existing information on mitotic hotspot identification. The criteria for defining hotspots are as follows: (1) hotspots are located at the most cellular areas of the tumor (more basophilic areas with nuclear overlapping and increased tumor cell density relative to other areas); (2) hotspots are frequently located at the peripheral invasive part of the tumor, but they can be central or scattered throughout the tumor; and (3) hotspots are more frequent in areas with solid growth pattern, sheets, and nested architecture than in areas with tubular or single cell infiltrative pattern.

These defining criteria were applied to test for improvement in interobserver concordance on hotspot identification (Table 3).

Interobserver agreement on choosing the same hotspot was tested before using these refined criteria for hotspot identification on WSI and it showed a moderate interobserver agreement (kappa = 0.53). An improvement of interobserver agreement on choosing the same hotspots was observed after applying these criteria (kappa = 0.75).

### Mitotic counts in hotspots versus non-selected areas of similar size

**Fig. 4:**
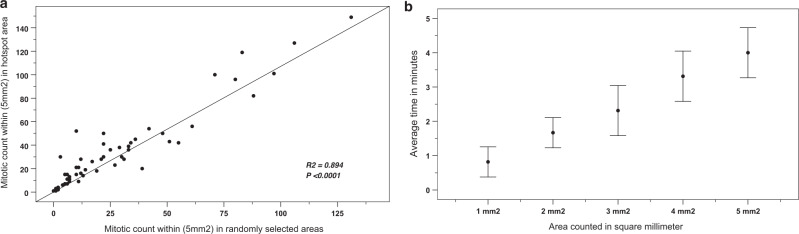
The correlation between mitotic count in random and hotspot areas, and the average time taken in minutes to count mitotic figures in different areas. **a** Scatter plots, showing the correlation of mitotic count within (5 mm^2^) area in hotspot and randomly selected areas and the tendency to underestimate mitotic counts in randomly selected areas, X-axis represents mitotic count within (5 mm^2^) randomly selected areas, and Y-axis represents mitotic count within (5 mm^2^) in hotspot areas. **b** A plot showing the relation between the average time taken in minutes (mean ± SD) to count mitotic figures in different areas (1, 2, 3, 4, and 5 mm^2^) using WSIs (Case Viewer software), X-axis represents different area sizes, and Y-axis represents time taken in minutes.

The median mitotic counts in a defined area within selected hotspots were higher than the median mitotic count in the same size of the randomly selected areas of the same size and the difference was statistically significant (*P* < 0.001). Figure 4a shows the relationship (scatter plots) of mitotic count between hotspot and randomly selected areas. Moreover, the median mitotic counts in hotspots per mm^2^ were significantly higher than the median mitotic count per mm^2^ when mitotic figures were scored in the whole slide and the difference was statistically significant *P* < 0.001 (Supplementary Fig. S4).

### The optimal area of counting mitoses

Supplementary Table S3 shows the mean, median, 95% confidence interval for the mean, SD, and variance of mitotic count in different areas, while Supplementary Fig. S4 boxplot shows the median, minimum, and maximum range of average mitotic count (mitotic count/area) in different areas within the hotspots.

**Fig. 5:**
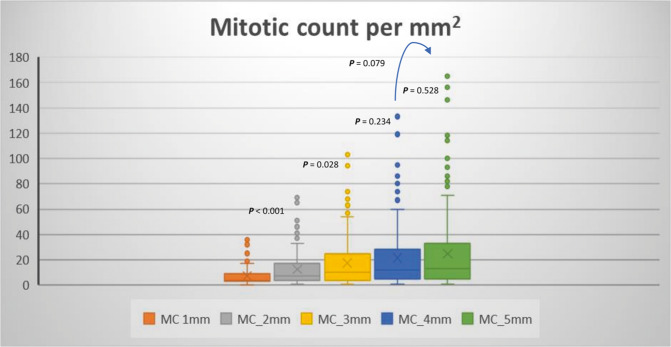
Boxplot showing median, minimum, maximum, and interquartile range of mitotic count in 1, 2, 3, 4, and 5 mm^2^ within the mitotically active areas. The figure shows a statistically significant difference between the median count in these areas (Mann–Whitney *U* test). X-axes represent mitotic figures, while Y-axis represent different areas (1, 2, 3, 4, and 5 mm^2^). Counting in 1 and 2 mm^2^ showed a statistically significant difference in the median count (*P* < 0.05). while counting in areas larger than 3 mm^2^ did not show a significant statistical difference. *P* = 0.234 between 3 and 4 mm^2^, *P* = 0.528 between 4 and 5 mm^2^, or *P* = 0.079 between 3 and 5 mm^2^.

Counting mitoses in 1 and 2 mm^2^ showed a statistically significant difference in median counts (*P* < 0.05). We found that counting in areas larger than 3 mm^2^ does not have a significant statistical difference (level of saturation) in the median count when comparison was run between 3 and 4 mm^2^ (*P* = 0.234), between 4 and 5 mm^2^ (*P* = 0.528), or between 3 and 5 mm^2^ (*P* = 0.79) (Fig. 5).

The highest degree of interobserver agreement among three pathologists was observed when counting mitoses in 3 mm^2^ (ICC = 0.691), while counting in 1 mm^2^ had the lowest interobserver agreement (ICC = 0.595) (Supplementary Table S4).

The average time (mean ± SD) for counting mitotic figures using WSI is illustrated in Fig. 4b, which shows a progressive increase in time from 1 mm^2^ (50 ± 30 s) to 5 mm^2^ (240 ± 50 s).

Survival analysis using the univariate cox regression model showed that mitotic counts in 2, 3, 4, and 5 mm^2^ areas were associated with BC-specific survival ((HR = 1.025, *P* = 0.02), (HR = 1.018, *P* = 0.016), (HR = 1.014, *P* = 0.009), and (HR = 1.012, *P* = 0.007), respectively), while mitotic count in 1 mm^2^ did not show an association with outcome (HR = 1.032, *P* = 0.134) (Supplementary Fig. S5).

Table 4 shows a constellation of factors for each area size and justifying the choice of the optimal area size of 3 mm^2^.

**Table 4:** Criteria applied for selection of the optimal area size.

Parameter	1 mm^2^	2 mm^2^	3 mm^2^	4 mm^2^	5 mm^2^
Level of saturation (i.e., counting mitosis in an area is not significantly different from the subsequent wider area) (Mann–Whitney *U* test)	X	X	√	√	√
	**(*P*** < 0.05)	**(*P*** < 0.05)	**(*P*** > 0.05)	**(*P*** > 0.05)	**(*P*** > 0.05)
Interobserver concordance level (inter-cluster correlation coefficient (ICC))	X	√	√	√	√
	(ICC = 0.595)	(ICC = 0.667)	(ICC = 0.691)	(ICC = 0.675)	(ICC = 0.686)
Time effective, s (mean ± SD)	√	√	√	X	X
	50 ± 30	100 ± 30	140 ± 50	200 ± 50	240 ± 50
Comparable to commonly used microscopes	X	√	√	X	X
Association with the outcome (BCSS) Cox regression test	X	√	√	√	√
	(*P* = 0.134)	**(*P*** = **0.021)**	**(*P*** = **0.016)**	**(*P*** = ***0.009*)**	**(*P*** = **0.007)**

Statistically significant *p*-values are in bold

“X” refers to lower performing or unreliable criterion.

“√” refers to higher performing or more reliable criterion.

### Methods of navigating WSIs when counting mitoses

To assess the best approach for counting mitoses on WSI, we compared three counting methods utilizing the same area (3 mm^2^): multiple HPSFs (×40), pre-annotated single area, and multiple areas. We found that the median mitotic count using multiple HPSFs that were calculated to produce 3 mm^2^ (i.e.,15 full monitor screen at ×40, using 27-inche monitor, BenQ, 2560 × 1440 pixels) was higher than counting within multiple or single annotated rectangles of the same area size and the difference was statistically significant (*P* < 0.001) (Table 5).

**Table 5:** The median mitotic count, while assessing the same area (3 mm^2^) using (multiple digital high-power (×40) screen fields (HPSF), multiple and single annotated rectangle), utilizing 27 inches, 2560 × 1440 pixels monitor (*n* = 132).

	Multiple digital high-power (×40) screen fields (HPSF)	Multiple separate annotated rectangles measuring 3 mm^2^	Single annotated rectangle measuring 3 mm^2^
Mean	21.2	19.3	16.1
Median	14	12	9
Minimum	0	0	0
Maximum	149	126	105

Underlined values represent the median mitotic count.

When using multiple HPSFs, the interobserver agreement between two observers was higher compared to using multiple annotated and single annotated rectangles (ICC = 0.911, ICC = 0.877, and ICC = 0.840, respectively). In addition, we found that using the multiple HPSFs was more practical (timesaving) (Table 6) as it did not require annotation on the WSI or consideration for counting fields within the annotated areas to avoid overlaps with counting the same mitotic figures or missing some mitotic figures in between fields.

**Table 6:** Interobserver agreement in counting mitoses in the defined area, correlation with the original mitotic score, and average time using different counting methods (multiple digital high-power (×40) screen fields (HPSF), multiple and single annotated rectangle) of the same areas 3 mm^2^ (27-inch, 2560 × 1440 pixels).

	Multiple digital high-power (×40) screen fields (HPSF)	Multiple separate annotated rectangles	Single annotated rectangle
Inter-cluster correlation coefficient (ICC)	0.911	0.877	0.84
Correlation with the original mitotic score (Fleiss’ Kappa)	0.478	0.431	0.344
Average time per case (s)	110 ± 30	150 ± 30	140 ± 30
Number	50	50	50

### Factors affecting size displayed on digital platforms

As HPSF showed the best method for counting mitoses, it is important to standardize the number of screen fields required to measure a defined area (e.g., 3 mm^2^). Therefore, we tested the impact of several factors on the size of the screen field. We found that the monitor screen resolution is the most important factor.

**Fig. 6:**
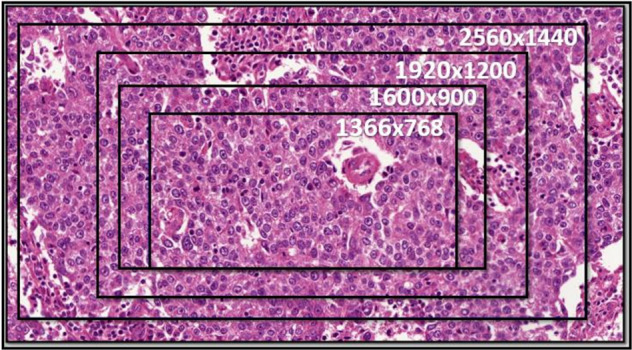
The effect of changing the resolution (in pixels) on the area size of a fixed 27-inch monitor (Case Viewer software). On the same tumor focus, utilizing (case-viewer image software) at ×40 digital magnification displaying different resolutions while using the same monitor size (27 inches, BenQ GW2765).

Figure 6 shows the effect of different resolutions on the area size displayed on monitors of the same size (27 inches).

**Fig. 7:**
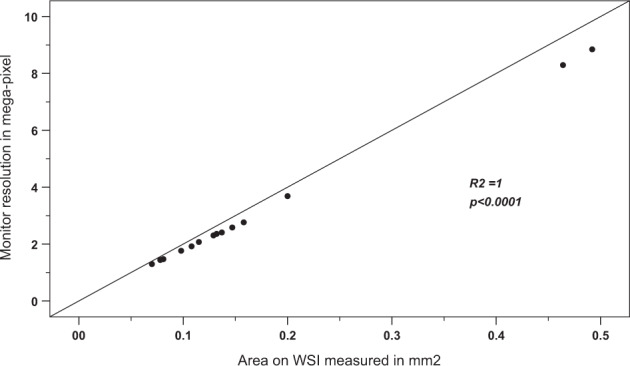
The effect of changing the resolution on the area on WSI using a fixed 27-inch monitor 3DHISTECH (Case Viewer software). Scatter plots and regressions describing the relation between the monitor resolution and the area on WSI. The size of the area on the monitor at ×40 digital magnification changes proportionately and significantly with a linear relationship, X-axis represents area on WSIs in mm^2^, and Y-axis represents monitor resolution in megapixels.

We found that while fixing all the variables and changing only the display resolution, the size of the area on the monitor changed proportionately and significantly with a linear relationship (Fig. 7).

When the monitor screen resolution is fixed, the monitor screen size resulted in an insignificant change in the size of the area.

The monitor screen size had an almost negligible impact on the field size and subsequently, the number of HPSF used to cover the defined area.

When scanning slides by two different scanners using the same image viewing software, we found that the area size changed, confirming the effect of the scanner’s camera sensor and resolution on the overall viewing area size (Supplementary Fig. S6).

Accordingly, our results confirmed that the size of the viewing area displayed on the monitor at the same magnification (×40) varies with screen resolution, scanner type used, and viewing software, while the monitor size does not have any significant effect. With the available data, we were able to produce the equation below to determine the number of HPSFs (the area size on the screen at ×40, which can be multiplied to produce 3 mm^2^ area). Since slides are typically scanned at a fixed magnification (×40), the digital camera’s sensor resolution, monitor resolution as well as viewing software were the changing variables.Equation: (a1 × scanner camera resolution + a2 × monitor resolution + a3 × software viewing area) = WSI viewer area.

The relationship between variables can be described using this equation, where a1, a2, and a3 are constants that control how much these elements contribute to the viewer area.

Some viewing software features built-in algorithms for determining the area displayed on screen. Table 7 shows different monitor sizes with different resolutions and the number of HPSFs needed to account for area size of 2 and 3 mm^2^, respectively.

**Table 7:** Details of the area size and number of full screens to cover defined area while counting mitosis using common monitor sizes and resolution.

Monitor sizes (inches)	Aspect ratio	Resolution (pixels)	Resolution (megapixels)	WSI viewer area (mm^2^)	WSI Viewer software	×40 digital HPSF nearly equivalent to 3 mm^2^	×40 digital HPSF nearly equivalent to 2 mm^2^	Size area of 10 digital ×40 HPSF
32” Samsung LU32J590UQUXEN	16:09	3840 × 2160	8.29	0.460	Case Viewer (3DHISTECH)	7 (3.2 mm^2^)	5 (2.3 mm^2^)	4.6 mm^2^
	16:09	3840 × 2160		0.436	IMS (Philips)	7 (3.1 mm^2^)	5 (2.2 mm^2^)	4.4 mm^2^
	16:09	3840 × 2160		0.517	Aperio (Leica)	6 (3.1 mm^2^)	4 (2.1 mm^2^)	5.2 mm^2^
27” BenQ GW2765	16:9	2560 × 1440	3.68	0.209	Case Viewer (3DHISTECH)	15 (3.1 mm^2^)	10 (2.1 mm^2^)	2.1 mm^2^
	16:09	2560 × 1440		0.193	IMS (Philips)	16 (3.1 mm^2^)	11 (2.1 mm^2^)	1.9 mm^2^
	16:09	2560 × 1440		0.230	Aperio (Leica)	13 (3.0 mm^2^)	9 (2.1 mm^2^)	2.3 mm^2^
27” Dell U2719D	16:09	1600 × 900	1.44	0.078	Case Viewer (3DHISTECH)	39 (3.0 mm^2^)	26 (2.0 mm^2^)	0.8 mm^2^
	16:09	1600 × 900		0.067	IMS (Philips)	45 3.0 mm^2^)	30 (2.0 mm^2^)	0.7 mm^2^
	16:09	1600 × 900		0.088	Aperio (Leica)	35 (3.1 mm^2^)	23 (2.0 mm^2^	0.9 mm^2^
24” Samsung S24E450B	16:09	1920 × 1080	2.07	0.115	Case Viewer (3DHISTECH)	26 (3.0 mm^2^)	18 (2.1 mm^2^)	1.2 mm^2^
	16:09	1920 × 1080		0.102	IMS (Philips)	30 (3.1 mm^2^)	20 (2.0 mm^2^)	1.0 mm^2^
22” iiyama P2252HS-B1	16:09	1920 × 1080	2.07	0.115	Case Viewer (3DHISTECH)	26 (3.0 mm^2^)	18 (2.1 mm^2^)	1.2 mm^2^
	16:09	1920 × 1080		0.102	IMS (Philips)	30 (3.1 mm^2^)	20 (2.1 mm^2^)	1.0 mm^2^
	16:09	1920 × 1080		0.128	Aperio (Leica)	24 (3.1 mm^2^)	16 (2.0 mm^2^)	1.3 mm^2^
20” HPLA2006	16:09	1600 × 900	1.44	0.078	Case Viewer (3DHISTECH)	39 (3.0 mm^2^)	26 (2.0 mm^2^)	0.8 mm^2^
	16:09	1600 × 900		0.067	IMS (Philips)	45 (3.0 mm^2^)	30 (2.0 mm^2^)	0.7 mm^2^
19” iiyama ProLite E1980SD	05:04	1280 × 1024	1.31	0.072	Case Viewer (3DHISTECH)	42 (3.0 mm^2^)	28 (2.0 mm^2^)	0.7 mm^2^
	05:04	1280 × 1024		0.063	IMS (Philips)	48 (3.0 mm^2^)	32 (2.0 mm^2^)	0.6 mm^2^
	05:04	1280 × 1024		0.081	Aperio (Leica)	38 (3.1 mm^2^)	25 (2.0 mm^2^)	0.8 mm^2^
17” DELL 210-AEUR	04:03	1280 × 1024	1.31	0.072	Case Viewer (3DHISTECH)	42 (3.0 mm^2^)	28 (2.0 mm^2^)	0.7 mm^2^
	04:03	1280 × 1024		0.063	IMS (Philips)	48 (3.0 mm^2^)	32 (2.0 mm^2^)	0.6 mm^2^

## Discussion

The introduction and implementation of WSI in routine clinical settings for primary diagnosis have brought some potential benefits as well as some challenges, although WSI provides the advantage of an enhanced low-power overview of the slide and allows the integration of diagnostic AI algorithms.

Studies on mitoses detection using AI have been published^37–39^. However, the consistency in the methodology and definitions of the mitotic index or score remain lacking, which makes it difficult to be integrated in the final Nottingham histological grade. The current study aimed to provide evidence of the methodology and a protocol that can improve concordance using WSIs and to guide future AI-based mitosis detection studies in refining performance and improving consistency of reporting.

The mitotic hotspots are most representative of the proliferative capacity of the tumor^9^. Our results showed that there is a tendency to underestimate mitotic count in randomly selected areas.

In the present study, we revealed that mitotic figures in the majority of cases show regional variation either located at the periphery or situated at the center, suggesting that this variation in the distribution of mitotic figures can be considered as a major cause of interobserver variability. Using WSIs, we searched for clues to attain better agreement in choosing mitotic hotspots, to refine the existing criteria, and to provide evidence-based data on identifying hotspots. Relative increase in tumor cell density is the easiest way to find mitotic hotspots regardless of distribution. Assessment of tumor for foci of higher cellularity relative to surrounding areas at the very low power available can provide better agreement on identifying hotspots. Moreover, hotspots distribution was different for intrinsic subtypes and peripheral distribution was associated with TNBC. Previous studies have reported that TNBC with acellular zone is associated with worse prognosis and higher risk of lung and brain metastases^40–42^.

It was recently suggested that a predefined region of the tissue measured in mm^2^ is favored over methods focusing on the number of HPFs when using a microscope^43^, and some studies found that counting mitoses per mm^2^ have better reproducibility than per HPFs^16,21,22^. However, the practice in counting mitoses in BC remains dependent on the use of ten HPFs and estimating the size of the area of these ten HPFs rather than using a variable number of HPFs to achieve a defined area (e.g., 2 or 3 mm^2^). In this study, we tested several area sizes to identify the optimal area; however, we did not exceed 5 mm^2^ as larger areas are believed to be impractical^20^, time-consuming, and represent more than any area covered by ten HPFs even with the widest ×40 fields on conventional microscope lenses currently available in the clinical setting. Our findings showed that 1 mm^2^ is not reliable for counting mitoses as it is associated with lower mitotic count and low concordance; in addition, it did not show significant association with the patient outcome. While 2 mm^2^ was the smallest area associated with patient outcome, mitoses counting in 3 mm^2^ appeared to be more representative when considering the level of saturation, scoring time, and reproducibility; there was no statistically significant impact on scoring when compared to a wider area. It is worth mentioning that 3 mm^2^ area is comparable to ten HPF of a high field diameter microscope (0.62 mm), which we currently use in our clinical practice. If we convert these assessed factors into acquired points, we can see that 3 mm^2^ achieved the highest points. Consistent with our finding, Meyer et al.^20^ showed that less sampling error is associated with larger sample areas and this has a non-linear relationship with the size of the area to be sampled, where a slight increase in the area has a significant effect on reducing the misclassification rate. This fits more in tumors with a low mitotic count, and the misclassification rates nearly reduced to half when increasing the area from 1.26 to 3.74 mm^2 ^^14^. The need to count mitoses in ≥2 mm^2^ to adjust the number of microscopes HPFs has also been stated in many previous studies^1,14,17,20^.

When testing several approaches for scoring mitoses on the WSI, we have found that using screen fields was the most reliable, timesaving, and practical compared with annotated areas. In this study, we suggest using the term digital HPSFs to be used in digital reporting instead of the conventional term HPF applied to microscopes. In this situation, high power (×40) refers to the scanner lens magnification and/or the magnification on the screen. Although some scanners can use a ×20 magnification lens but the ×40 is the standard. In this study, we used a ×40 lens and ×40 magnification power on the screen. Higher magnification can be used to improve the visualization of mitotic figures but the standard mitoses counting on the defined area was performed on ×40 magnification.

Several variables could affect the size of the displayed area at ×40 magnification on WSIs. These are mainly the scanning resolution, the viewing monitor resolution, the image viewer software, and the type of scanner used^26^. We investigated the parameters influencing the size of the area displayed on the screen at ×40 digital magnification and discovered that monitor resolution has a substantial effect, whereas display size has an insignificant effect. It is worth noting that when scanning the same slide with different scanners at a fixed magnification while keeping and monitor screen resolution unchanged, there was an insignificant change in the size area of viewing, However, this is unlikely to be a noticeable issue if the same scanner is routinely used in a department. The viewing software can also have a limited effect due to window and toolbar size. Kim et al.^28^ addressed this point and found that the size of the area displayed on the screen at ×40 digital magnification is mainly affected by display resolution, and WSI viewer, but not by changing the scanner type or scanning resolution. Therefore, it is important to adjust the area of viewing on the screen to calculate the number of HPSFs that is needed to produce a defined area on WSI. A toggle to HPSF could be added to the viewer software may help in this regard. Table 6 shows the details of the area size and number of HPSF to cover a defined area, which is either 3 or 2 mm^2^ when counting mitosis using common monitor sizes and resolution. From the table, it is clear that ten HPSF produced variable areas and when using certain monitor screens, this area can be <2 mm^2^.

In a recent study, we showed that there is an average 20% reduction of mitotic counts on WSI compared to light microscopy^26^. Although this can be explained partially by the scanner and/or image quality, and lack of familiarity with WSI, we noted this reduction with most of the commercially available clinical grade tools without significant improvement with training. Therefore, a new cut-off of mitotic count for scores 1, 2, and 3 as a component of the Nottingham grade is needed. This should ideally be based on the level of reduction of the mitotic count, and the association with patient’s outcome utilizing a large well-established BC cohort with long term outcome data.

Finally, as we are approaching the approval for scanner use in primary diagnosis, the first objective would be to improve reproducibility and concordance in BC grading, as a result a proposed protocol for agreement on a standard area to count mitoses is a priority when using WSI. Standardizing the area using WSI, together with better recognition of mitotic figures will facilitate the adoption of WSI in routine practice. Counting mitoses in BC can reliably be done on WSIs in 3 mm^2^, ideally with an adjusted number of HPSF. The quality of the scanner’s digital camera and the monitor resolution are mostly responsible for delivering high-quality images and fewer HPSFs to cover 3 mm^2^ area, thereby we recommend using a high-resolution display monitor in routine practice to save time and produce more reliable BC grading. We believe these steps will provide the base evident approach that will be the cornerstone template to develop and validate robust AI-based tools to count mitoses in a standardized manner. They also provide a reliable stopgap for eyeballing assessment of mitoses until these AI algorithms are well validated and implemented.

## Supplementary information

Supplementary material

## Data Availability

The data that support the findings of this study are available from the corresponding author upon reasonable request.
