# Supplementary material for: Defining the area of mitoses counting in invasive breast cancer using whole slide image
Source: Mod Pathol. 2021 Dec 11;35(6):739–48. doi: 10.1038/s41379-021-00981-w (PMC9174050; doi:10.1038/s41379-021-00981-w)
Supplement: Supplementary file 1 — Supplementary material [file 41379_2021_981_MOESM1_ESM.docx]

**Supplementary Tables**

**Table S1. The morphological characteristics of the studied cases.**

| **Morphological characteristics of the studied cases** | **Number** | **(%)** |
| --- | --- | --- |
| **Central fibrosing scar** |  |  |
| Presence of central fibrous scar | 43 | (38.1) |
| Absence of central fibrous scar | 70 | (61.9) |
| **Central necrosis** |  |  |
| Presence of necrosis | 16 | (14.2) |
| Absence of necrosis | 97 | (85.8) |
| **Tumor borders** |  |  |
| Infiltrative | 101 | (89.4) |
| Pushing | 12 | (10.6) |
| **Presence of TILS** |  |  |
| Mild | 99 | (87.6) |
| Moderate | 8 | (7.1) |
| Marked  **Tumor Pattern of growth** | 6 | (5.3) |
| Tubular | 16 | (14.2) |
| Single files | 11 | (9.7) |
| Sheets | 38 | (33.6) |
| Nests and trabeculae | 45 | (39.8) |
| papillary | 2 | (1.8) |
| Cribriform | 1 | (0.9) |

**Table S2. The relation between hotspot distribution with other morphological parameters.**

|  |  | **Distribution of Hotspot** | | |  | **Total** | ***x^2^***  ***P***-value |
| --- | --- | --- | --- | --- | --- | --- | --- |
|  |  | **Peripheral** | **Scattered** | **Central** | **No hotspot** |  |  |
| **Relative increase in tumor cell density** | Present  Absent | 35 (39.8)  4 (16) | 45(51.1)  9 (36) | 5 (5.7)  4 (16) | 3(3.4)  8(32) | 88  25 | 23.07  **P <0.001** |
| **Pattern** | Tubular  single files  Sheets  Nests & trabeculae  Papillary  Cribriform | 7(43.8)  2(18.2)  15(39.5)  15(33.3)  0(0)  0(0) | 4(25)  2(18.2)  21(55.3)  26(57.8)  0(0)  1(100) | 3(18.8)  3(27.3)  1(2.6)  2(4.4%)  0(0)  0(0) | 2(12.5)  4(36.4)  1(2.6)  2(4.4)  2(100)  0(0) | 16  11  38  45  2  1 | 108.56  **P <0.001** |
| **Mitotic count within the hotspot area** | ≤ 6  7-22  ≥23 | 3(11.5)  22(40.7)  14(42.4) | 6(23.1)  30(55.6)  18(54.5) | 6(23.1)  2(3.7)  1(3) | 11(42.3)  0(0)  0(0) | 26  54  33 | 56.0  **P <0.001** |
| **Central fibrosis** | Present  Absent | 33 (76.7)  6(8.6) | 7 (16.3)  47(67.1) | 1(2.3)  8 11.4) | 2(4.7)  9(12.9) | 43  70 | 54.9  **P <0.001** |
| **Central necrosis** | Present  Absent | 10(62.5)  29(29.9) | 5(31.3)  49(50.5) | 0(0)  9(9.3) | 1(6.3)  10(10.3) | 16  97 | 7.75  P =0.05 |
| **Tumor border** | Infiltrative  Pushing | 38(37.6)  1(8.3) | 47(46.5)  7(58.3) | 8(7.9)  1(8.3) | 8(7.9)  3(25) | 101  12 | 6.195  P=0.103 |
| **^*^ TILS** | Mild  Moderate  Marked | 35(35.4)  1(12.5)  3(50) | 44(44.4)  7(87.5)  3(50) | 9(9.1)  0(0)  0(0) | 11(11.1)  0(0)  0(0) | 99  8  6 | 7.26  P = 0.146 |

***p value in bold: significant***

***^*^ TILS (Tumour infiltrating lymphocytes).***

**Table S3.** **The mean, median, 95% confidence interval for the mean, standard deviation (SD) and variance of mitotic count (MC) per different hotspot areas size (N=160).**

| Area size | Mean MC | 95 % confidence interval of the mean MC | | Median MC | SD | Variance |
| --- | --- | --- | --- | --- | --- | --- |
|  |  | **Lower Bound** | **Upper Bound** |  |  |  |
| 1mm2 | 8.9 | 7.37 | 10.47 | 6 | 8.3 | 69.538 |
| 2mm2 | 15.7 | 13.02 | 18.52 | 11 | 14.759 | 217.822 |
| 3mm3 | 22.4 | 18.37 | 26.43 | 15 | 21.635 | 468.081 |
| 4mm4 | 27.47 | 22.31 | 32.63 | 18 | 27.679 | 766.126 |
| 5mm5 | 31.7 | 25.51 | 37.89 | 21 | 33.215 | 1103.212 |

**Table S4. Inter-observer agreement of mitotic count using (ICC) in 1, 2, 3, 4 and 5mm^2^.**

| Area | Agreement (ICC) among three observers, 95%(CI) |
| --- | --- |
| 1mm2 | 0.595 (0.420-0.743) |
| 2mm2 | 0.667 (0.510-0.794) |
| 3mm2 | 0.691 (0.541-0.811) |
| 4mm2 | 0.675(0.520-0.799) |
| 5mm2 | 0.686 (0.532-0.808) |

**Supplementary Figures**

**Figure S1.** Showing various patterns of tumor growth assessed.


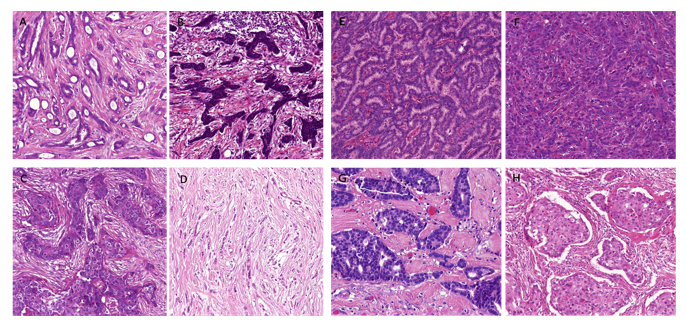


**Photomicrographs of various histological patterns of Haematoxylin and eosin (H&E) stained slides using WSIs at (20x) digital magnification.**

**(A)** The tumor is arranged in tubular pattern. the tumor is highlighted by irregularly distributed rounded and angulated tubules with open laminae. The tubules are lined by a single layer of epithelial cells and are surrounded by desmoplastic stroma

**(B)& (C)** The tumor shows trabecular pattern.

**(D)** lobular carcinoma: the tumor shows uniform tumor cells mostly arranged in a single file pattern.

**(E)** Shows papillary pattern.

**(F)** The tumor cells are arranged in sheets.

**(G)** The tumor cells are arranged in cribriform pattern.

**(H)** The tumor shows nested pattern.

**Figure S2.** WSIs showing inter-observer agreement and disagreement on choosing mitotic hot spot area.


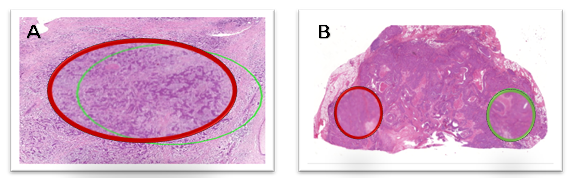


Two pathologists, each annotated the mitotic hot spot using the circle annotation tool equal to 5mm2, (one red circle, the other green circle).

**4A**: Shows agreement between observers on the same hotspot (nearly overlap) digital 2x magnification.

**4B**: Shows dis-agreement between observers on the hotspot location (digital 0.2x magnification).

**Figure S3.** The median mitotic counts in the hot spot area versus the whole slide per mm^2^.


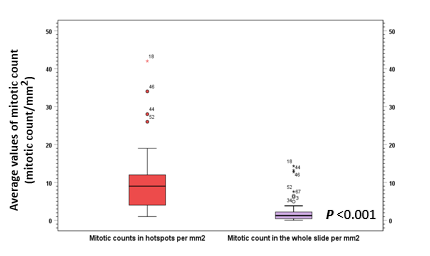


In the box plots, a black line within the box marks the median.

There is a statistically significant difference between the median count in WSI and Hotspot areas (*Mann-Whitney U test*).

**Figure S4.** Box plot depicting the median minimum and maximum range of average values of mitotic count (mitotic count/area) in different areas (1,2,3,4 and 5 mm^2^).


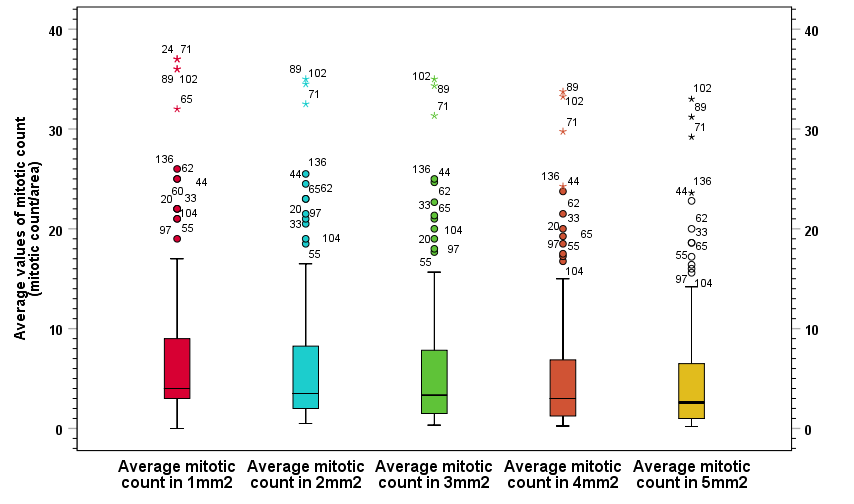


In the box plots, Whiskers above and below the box indicate the 10 ^th^ and 90 ^th^ percentiles. Points above and below the whiskers indicate outliers outside the 10 ^th^ and 90 ^th^ percentiles.

The median of the average count was highest when mitoses were counted in 1 mm^2^, with close but lower values when counted in 2, 3, 4 and 5 mm^2^.

**Figure S5.** A plot showing the relationship between the significance of association with breast cancer-specific survival (BCSS) ***P***-value against mitotic count in each area assessed(Cox regression test).


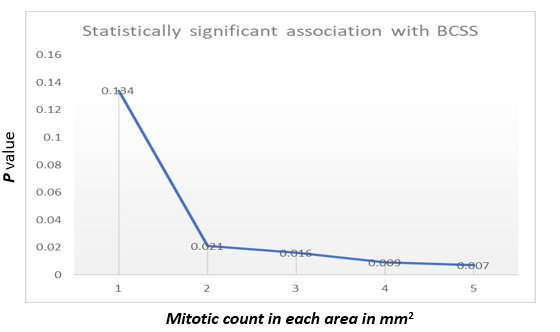


Mitotic counts in 2, 3, 4 and 5 mm^2^ showed a significant association with BCSS (***P*** = 0.021, ***P***= 0.016, ***P***= 0.009 and ***P*** = 0.007). respectively), while mitotic count in 1 mm^2^ did not show statistically significant association (***P*** = 0.134), (*Cox regression test).*

**Figure S6.** The Metadata of each WSI scanned by different scanners.


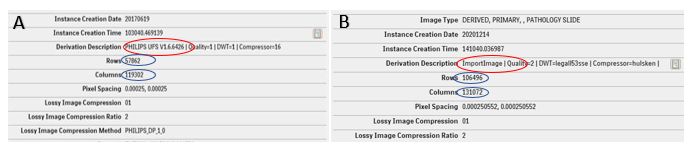


**A**: Metadata of slide scanned by *Philips* scanner and viewed by *Philips IMS*

**B:** Metadata of slide scanned by 2^nd^ party scanners and viewed by *Philips IMS. note* we get different size data.

***Red circles***: refer to the scanner type, blue ***circles:*** refer to metadata (rows and columns).
